# The effects of preoperative rehabilitation on pain and functional outcome after total knee arthroplasty: a meta-analysis of randomized controlled trials

**DOI:** 10.1186/s13018-022-03066-9

**Published:** 2022-03-21

**Authors:** Wanying Su, Yang Zhou, Hailing Qiu, Hui Wu

**Affiliations:** 1 Joint Surgery and Sport Medicine Department, Hunan Provincial People’s Hospital, The First Affiliated Hospital of Hunan Normal University, Hunan, China; 2 Department of Clinical Nursing, Xiangya Hospital of Central South University, Hunan, China

**Keywords:** Rehabilitation, Total knee arthroplasty, Meta-analysis, Randomized controlled trials

## Abstract

**Background:**

There have been controversial findings for the effectiveness of rehabilitation before operation after total knee arthroplasty (TKA). This study aimed to conduct an updated, comprehensive systematic review. On that basis, the review was to be combined with meta-analysis to measure the effects of rehabilitation before operation on functions and pain after TKA.

**Methods:**

Articles were searched by using Central Register of Controlled Trials (CENTRAL), Web of Science, EMBASE, Cochrane, Pubmed, CNKI, Wanfang, Weipu and the Chinese Biomedical Database from the beginning to December 10, 2021. The major outcomes included pain, knee flexion and extension, as well as knee range of motion (ROM). Secondary outcomes included timed-up-and-go (TUG), 6-min walk, and patient-reported functional outcome (the Knee Injury and Osteoarthritis Outcome Score (KOOS) or Western Ontario and McMaster Universities Osteoarthritis Index (WOMAC)). Third outcomes included the length of hospital stay.

**Results:**

Nineteen studies recruiting 1008 patients satisfied with the inclusion criteria. Significant difference was identified in knee flexion, TUG, KOOS (knee-associated life quality and functions in sports and recreation), as well as the length of hospital stay (*P* < 0.05). Insignificant statistical difference was identified in pain, 6-min walk, ROM, knee extension, KOOS (pain, symptoms and function of daily living) after TKA between the two groups. No difference was found between the groups in WOMAC.

**Conclusions:**

Preoperative rehabilitation could significantly shorten hospital stay, whereas there is not any conclusive evidence of the improvement of postoperative functions. Accordingly, in-depth high-quality studies should be conducted to confirm the effectiveness of preoperative rehabilitation in patients having received TKA.

**Supplementary Information:**

The online version contains supplementary material available at 10.1186/s13018-022-03066-9.

## Introduction

Osteoarthritis (OA) refers to a commonly used joint degenerative diseases, capable of leading to joint pain and disability. Total knee arthroplasty can effectively treat end-stage knee osteoarthritis, through which knee pain can be significantly effectively relieved, and knee function and quality of life of patients can be improved [1, 2]. 72,100 TKAs were reported in the United States in 2014, and the incidence of TKA was expected to increase from 78 to 182% in the period of 2014 to 2030 [3]. Though the hospitals have optimized many procedures to reduce patients' waiting time for TKA, whereas waiting times remain excessively long for patients with pain and disabilities [4]. Long time waiting may make the patient's muscle strength damage, reduce the range of motion, have negative consequences for postoperative outcome [5].

Appropriate rehabilitation after TKA may certainly affect the course and outcome of the surgery. Rehabilitation program mainly includes supervised rehabilitation and home-based programs. No matter which rehabilitation program can improve the postoperative function of knee surgery, so that patients get the best rehabilitation effect [6]. Preoperative training was reported as an effective and safe method to improve postoperative functional performance and muscle strength for patients having received TKA [7]. According to Calatayud et al. [8] high-intensity preoperative supervised training can reduce postoperative pain, improve lower limb muscle strength, range of motion, and shorten the length of hospital stay in patients with TKA. Moreover, Matassi et al. [9] reported that preoperative home exercise program is more conducive to the recovery of primary TKA patients, as opposed to the control. However, the effectiveness of preoperative rehabilitation is also uncertain. Huber et al. [10] reported that preoperative training programs did not benefit to postoperative functional recovery. Recently, Mat et al. [11] even reported that the 6-week preoperative physiotherapy did not significantly impact the early function and range of motion after TKA. Though existing systematically reviewing studies surveyed the effect of rehabilitation before operation on outcomes after the operation for patients having received TKA, there is still conflicting to whether preoperative rehabilitation improves postoperative outcome [12, 13]. There is a need to conduct a reviewing study the recent articles and assess the impact exerted by rehabilitation before operation on postoperative outcomes for patients having received TKA.

Thus, this study aimed to make a systematic review with an improved and extensive method. On that basis, the meta-analysis was combined to examine the impact of rehabilitation before operation on early functions and pain after TKA.

## Materials and methods

### Searching strategy and identification of literature

With Pubmed, Cochrane Central Register of Controlled Trials (CENTRAL), Web of Science, EMBASE, CNKI, Wanfang, Weipu and the Chinese Biomedical Database, the search was conducted from their inception to December 10, 2021. In addition, more articles were identified from relevant references. The search used was combined with subject words and free words (e.g., total knee replacement OR total knee arthroplasty OR TKA AND pre-habilitation OR rehabilitation OR resistance Training OR exercise OR training AND before operation). This study has been registered on the Research Registry, registration ID: reviewregistry1139.

### Inclusion and exclusion criteria

Inclusion criteria: (1) controlled articles under randomization. (2) Comparison of the preoperative rehabilitation group and the control who lived as usual. (3) Included patients were unilateral TKA with OA. (4) Clinical outcomes: the primary included pain, knee motion range (ROM), knee flexion and extension. Secondary outcomes included TUG, 6-min walk, patient-reported functional performance (KOOS or WOMAC). Third outcomes included the length of hospital stay.

Exclusion criteria: (1) Repeated published literature. (2) Not any outcome of interest suggested. (3) Trials type as “case reports”, “reviews”, “meta-analysis” and “letters”. (4) Animal experiments. (5) Articles without full text. (6) Documents not published in English or Chinese.

### Data extraction

All the literatures were imported into Endnote software, and the two investigators independently read abstracts and titles to preliminarily screen the articles under the inclusion and exclusion criteria. The literature that met the inclusion criteria was further read and screened again to determine whether it was included or not. Any objections to the included articles should be resolved on the basis of discussion or arbitration by a third investigator. The extraction contents include authors, publication year, country, patients, age, gander, body mass index (BMI), intervention measures and study type. After data extraction was completed, the two investigators cross-checked the extraction results. If the needed information in the article was missing or ambiguous, we attempt at contacting article authors for more details.

### Quality evaluation

Two investigating staffs conducted the independent evaluation of included trials quality by complying with the Cochrane Handbook for Systematic Reviews of Interventions guideline. Assessment indicators include selective reporting, incomplete outcome data, blinding of outcome assessment, blinding of participants and personnel, allocation concealment, random sequence generation, and other bias. The respective item had the evaluation to be "low risk bias”, “high risk bias" and "unclear". If the two investigators had different opinions in the process of inclusion literature and quality evaluation, they would discuss and resolve or request the third investigator to arbitrate.

### Data analysis and statistical method

The effect sizes of the respective analysis were determined using Review Manager Statistical software (version 5.3). Standardized mean difference (SMD) or weighted mean difference (WMD) acted as effects, the 95% confidence interval represents the effect size. The pooled odds ratio (OR) with 95% confidence intervals (95% CIs) was adopted to assist dichotomous results. Besides, the estimation of the uninterrupted results was made from the WMD or SMD pooled with 95% CIs. The estimation of the statistical heterogeneity between articles was made using the value of *P* and *I*^2^. A fixed-effect model was used when *P* > 0.1 and *I*^2^ < 50%; otherwise, a random-effect model was employed for the analyses. The sensitivity analysis was conducted to examine the likely heterogeneity source. Subsequently, the identified articles causing significant heterogeneity were excluded, and a repeated meta-analysis on the remaining articles was made for the adjustments. The meta-analysis robustness here was demonstrated if no considerable variations were being identified between the regulated and major results. The work has been reported in line with PRISMA (Preferred Reporting Items for Systematic Reviews and Meta-Analyses) and AMSTAR (Assessing the methodological quality of systematic reviews) Guidelines. PRISMA checklist is shown in the Additional file 1.

## Results

### Study selection and quality assessment

**Fig. 1:**
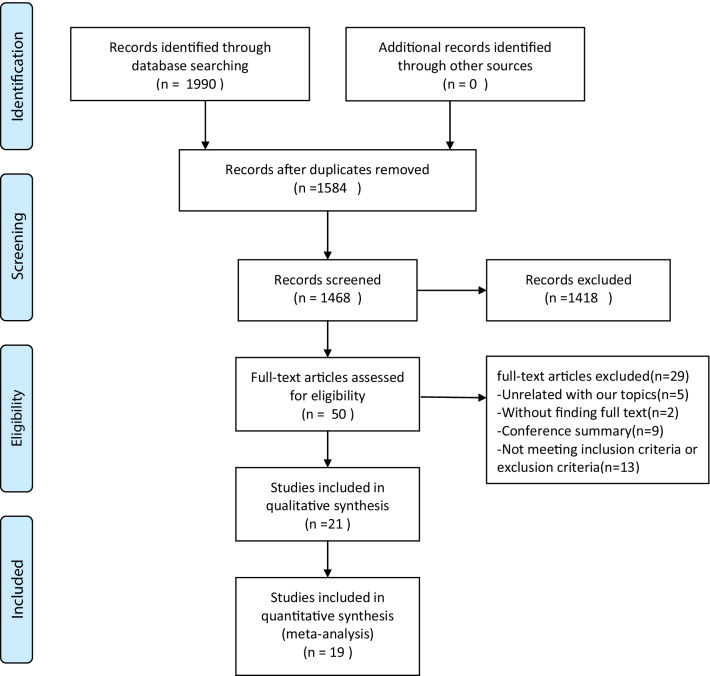
Flow chart of the systematic literature

**Fig. 2:**
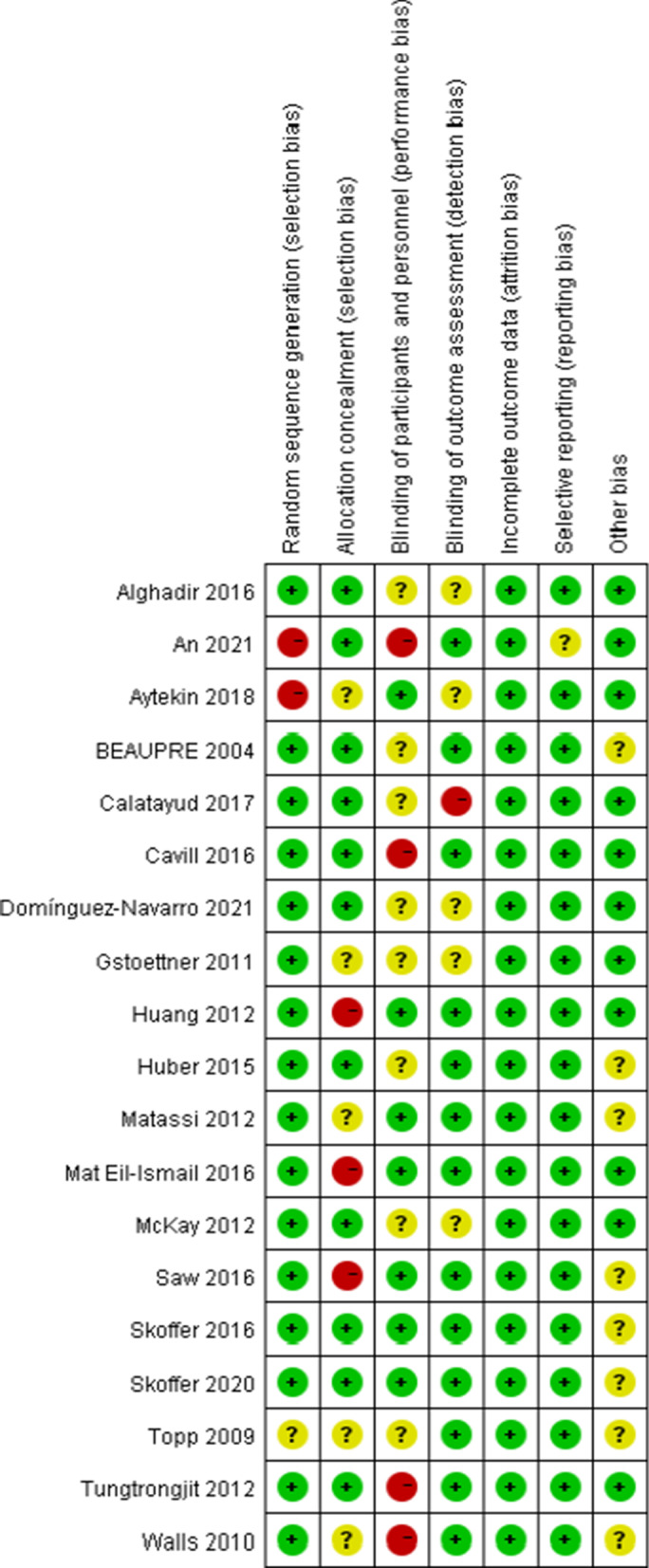
The risk of bias summary of the included studies

**Fig. 3:**
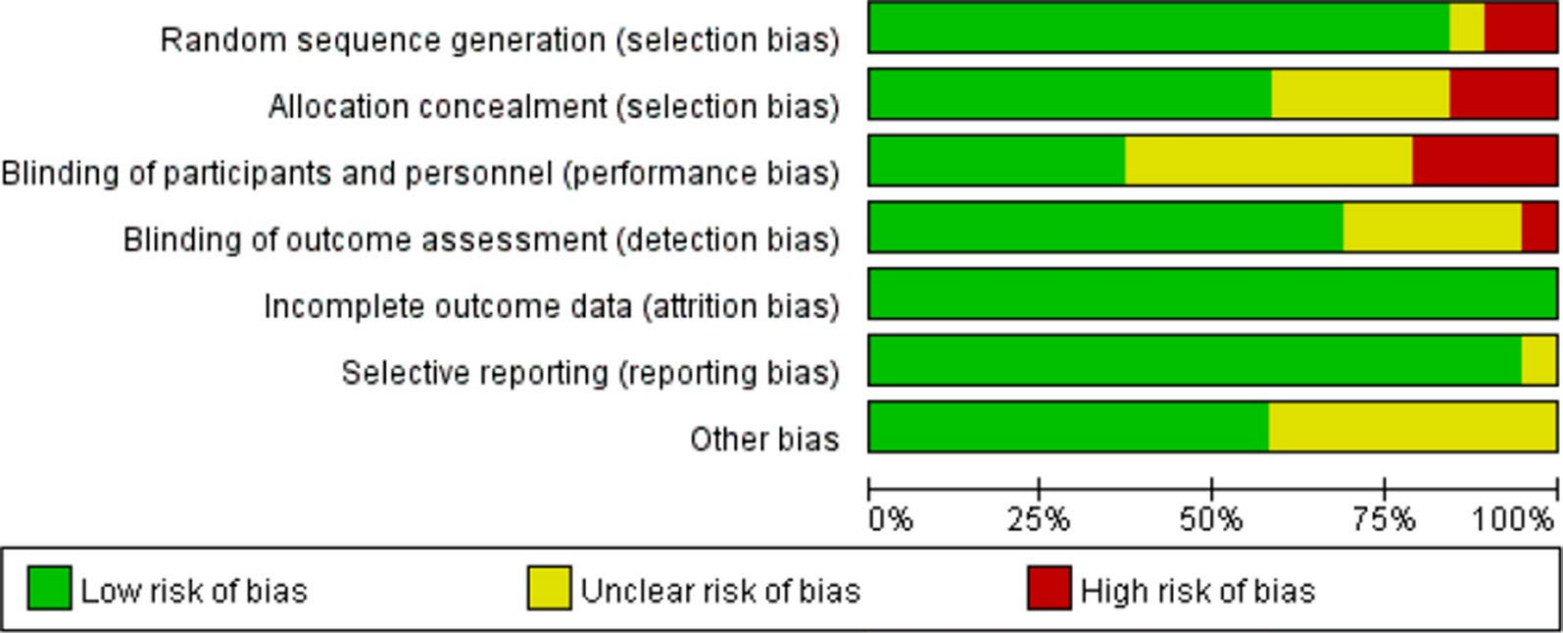
The risk of bias graph of the included studies

Following the existing searching strategy, 1990 studies originated in the online database from Jan. 1987 to Dec. 10, 2021. When duplicates were removed, 1584 studies were kept. Next, based on the view of titles and abstracts, 1418 articles were removed. Among the rest 50 studies, 31 studies were excluded, which was attributed to several factors. Lastly, 19 full-text articles were applicable to the present meta-analysis [8–11, 14–28] (Fig. 1). Table 1 and Figs. 2, 3 summarize the features, quality assessment and demographics of the articles included (Risk of bias summary).

**Table 1:** Characteristics of included studies

References	Country	Sample sizee, I/C	Age, I/C^†^ (year)	Female sex, I/C (%)	BMI (kg/m^2^)	Preop. intervention	Study type
Calatayud et al. [8]	Spain	25/25	66.8 ± 4.8/66.7 ± 3.1	84.1#	32.0 ± 4.2/31.0 ± 3.8	Seated leg press, knee extension, leg curl, and hip abduction (5 sets of 10 repetitions for each exercise, with 60 s rest between sets) 3 days per week for 8 weeks	RCT
Matassi et al. [9]	Italy	61/61	66 ± 7.2/67 ± 7.7	54.1/42.6	29.0 ± 4.3/28.0 ± 3.7	Muscle training: isometric quadriceps, isotonic hamstring, isotonic quadriceps contraction, and dynamic stepping exercise for 6 weeks	RCT
Huber et al. [10]	Switzerland	22/23	68.8 ± 8.0/71.9 ± 8.1	50.0/43.5	30.8 ± 4.9/29.9 ± 5.5	Neuromuscular training program for 4–12 weeks, depending on their location on the waiting list for surgery	RCT
Mat Eil-Ismail et al. [11]	Malaysia	24/26	62.4/64.3	91.7/80.8	–	Physical exercises (stretching, isometric strengthening exercises, mobilising exercises and heat therapy) for 6 weeks	RCT
Alghadir et al. [14]	India	25/25	63.3 ± 9.4^#^	58.2^#^	–	Strengthening and mobility exercises, proper techniques of transfers, and gait training, once a day for 30 min	RCT
Aytekin et al. [15]	Turkey	21/23	67.8 ± 6.3/69.7 ± 6.4	85.7/78.2	32.8 ± 5.9/30.2 ± 4.9	Education and home-based exercise, 2 sets of 10 repetitions of each exercise for five days/week for 12 weeks	RCT
Domínguez-Navarro et al. [16]	Spain	28/26	70.8 ± 5.4/70.4 ± 5.6	57.1/65.4	–	Strengthening training and progressive resistance exercise (the sessions lasted 30–40 min) for 5–8 weeks	RCT
Huang et al. [17]	Taiwan	126/117	69.8 ± 7.2/70.5 ± 7.4	69.8/73.5	27.1 ± 4.0/27.2 ± 4.5	Muscle strength training: knee setting, ankle pumping and hip abduction with resistance for 6 weeks	RCT
Saw et al. [18]	South Africa	35/39	60.7 ± 5.5^#^	81.1^#^	34.5 ± 8.2^#^	Six physiotherapist-led group-based sessions (two hours/week of education, exercise and relaxation)for 6 weeks	RCT
An et al. [19]	Korea	18/18	71.1 ± 3.3/70.4 ± 2.6	–	26.5 ± 2.5/26.5 ± 2.9	Preoperative telerehabilitation program (30 min/session, 2 times/day, 5 days/week for 3 weeks, for a total of 30 sessions)	RCT
Cavill et al. [20]	Australia	21/20	66.0 ± 8.4/68.3 ± 9.1	52.0/55.0	–	Prehabilitation included one-hour twice-weekly sessions for at least three and a maximum of 4 weeks prior to surgery	RCT
Skoffer et al. [21]	Denmark	30/29	70.7 ± 7.3/70.1 ± 6.4	63.3/58.6	30/31.8	Leg press, knee extension, knee flexion, hip extension, hip abduction, and hip adduction in strength training machines 3 training sessions per week for 4 weeks	RCT
Skoffer et al. [22]	Denmark	30/29	70.7 ± 7.3/70.1 ± 6.4	63.3/58.6	30/31.8	Leg press, knee extension, knee flexion, hip extension, hip abduction, and hip adduction in strength training machines 3 training sessions per week for 4 weeks	RCT
Walls et al. [23]	Ireland	9/5	64.4 ± 8.0/63.2 ± 11.4	67/80	30.7 ± 3.0/32.8 ± 6.3	8 weeks of preoperative unsupervised, home-based Neuromuscular electrical stimulation Straining applied unilaterally to the QFM of the affected side	RCT
McKay et al. [24]	Canada	10/12	63.5 ± 4.9/60.6 ± 8.1	50.0/66.7	35.0 ± 6.1/33.8 ± 7.1	A 10-min aerobic warm-up, followed by a circuit of bilateral lower body exercises (standing calf raise, seated leg press, leg curl, knee extension). 2 sets of 8 repetitions of each exercise	RCT
BEAUPRE et al. [25]	Canada	65/66	67.0 ± 7.0/67.0 ± 6.0	60.0/50.0	32.0 ± 6.0/32.0 ± 5.0	Crutch walking on level ground and on stairs, bed mobility and transfers, and the postoperative ROM routine, 3 times per week for 4 weeks for a total of 12 treatment sessions	RCT
Tungtrongjit et al. [26]	Thailand	30/30	63.0 ± 7.6/65.9 ± 7.2	86.7/80.0	24.3 ± 2.4/25.3 ± 3.8	The patients were asked to participate in 3 weeks Home Program (General Quadriceps strengthening exercise) until their TKA	RCT
Gstoettner et al. [27]	Austria	18/20	72.8 ± 15.7/66.9 ± 12.6	88.9/70	27.4/28.2	Preoperative proprioceptive training programme were taught and supervised for 45 min per setting by the same physical therapist for 6 weeks before TKA	RCT
Topp et al. [28]	America	26/28	64.1 ± 7.05/63.5 ± 6.68	73.1/64.3	32.16 ± 5.87/32.00 ± 6.09	Resistance, flexibility and step training, 1 supervised and 2 home sessions, 3 days per week for 4 weeks	RCT

I = intervention group, C =  control group

^†^Values are given as the mean with or without the standard deviation

^#^Patient demographics were not separated by randomized group

### Meta-analysis results

#### Visual analog scale (VAS)

**Fig. 4:**
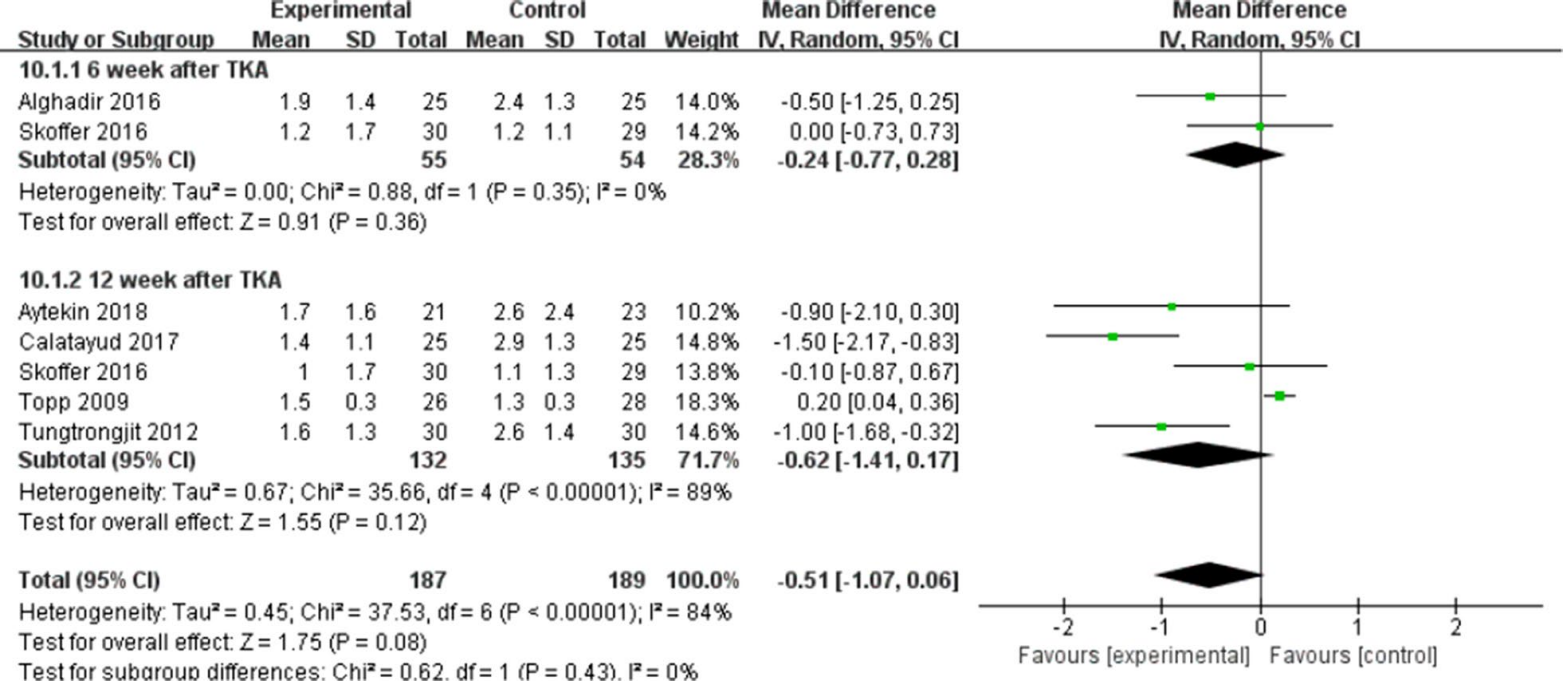
A forest plot diagram showing the VAS score

Pain was measured by VAS scores. Six studies [8, 14, 15, 21, 26, 28] (317 patients) reported the effects of pre-habilitation on postoperative pain. In this study, data were extracted twice. Subgroup analysis was conducted at the 6th and 12th weeks after TKA. As impacted by the heterogeneity of the subgroups (*I*^2^ = 84%, *P* = 0.08), the random response model was adopted. No significant difference received the identification between the two group [MD = − 0.51, 95% CI (− 1.07, 0.06), *P* = 0.08] (Fig. 4).

#### Knee motion range (ROM), knee flexion and extension

**Fig. 5:**
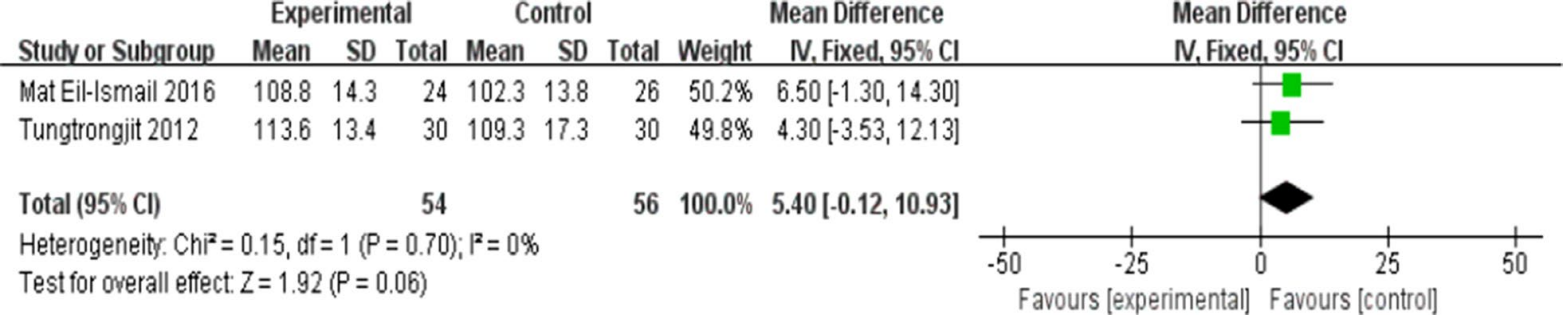
A forest plot diagram showing the ROM

**Fig. 6:**
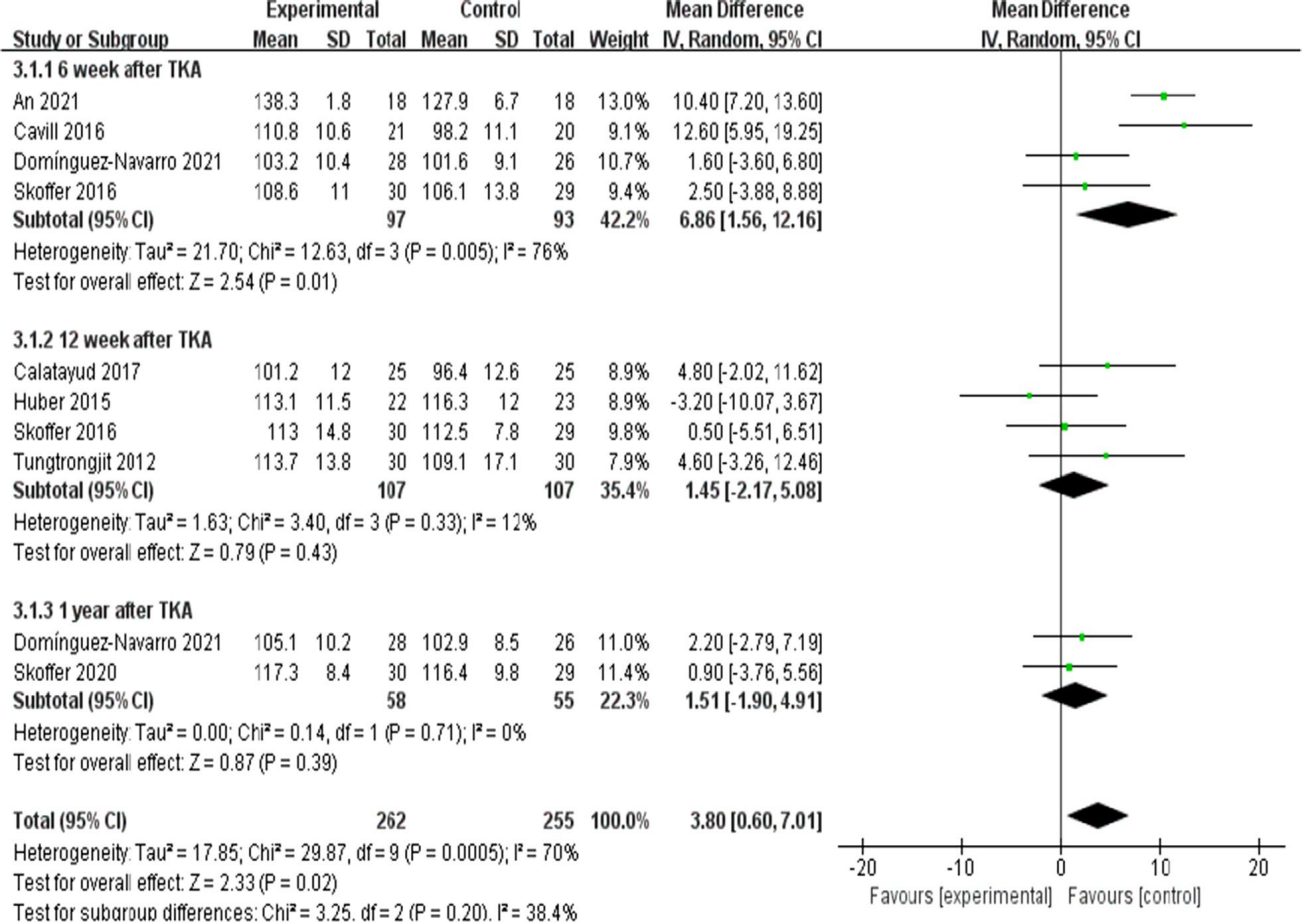
A forest plot diagram showing the knee flexion

**Fig. 7:**
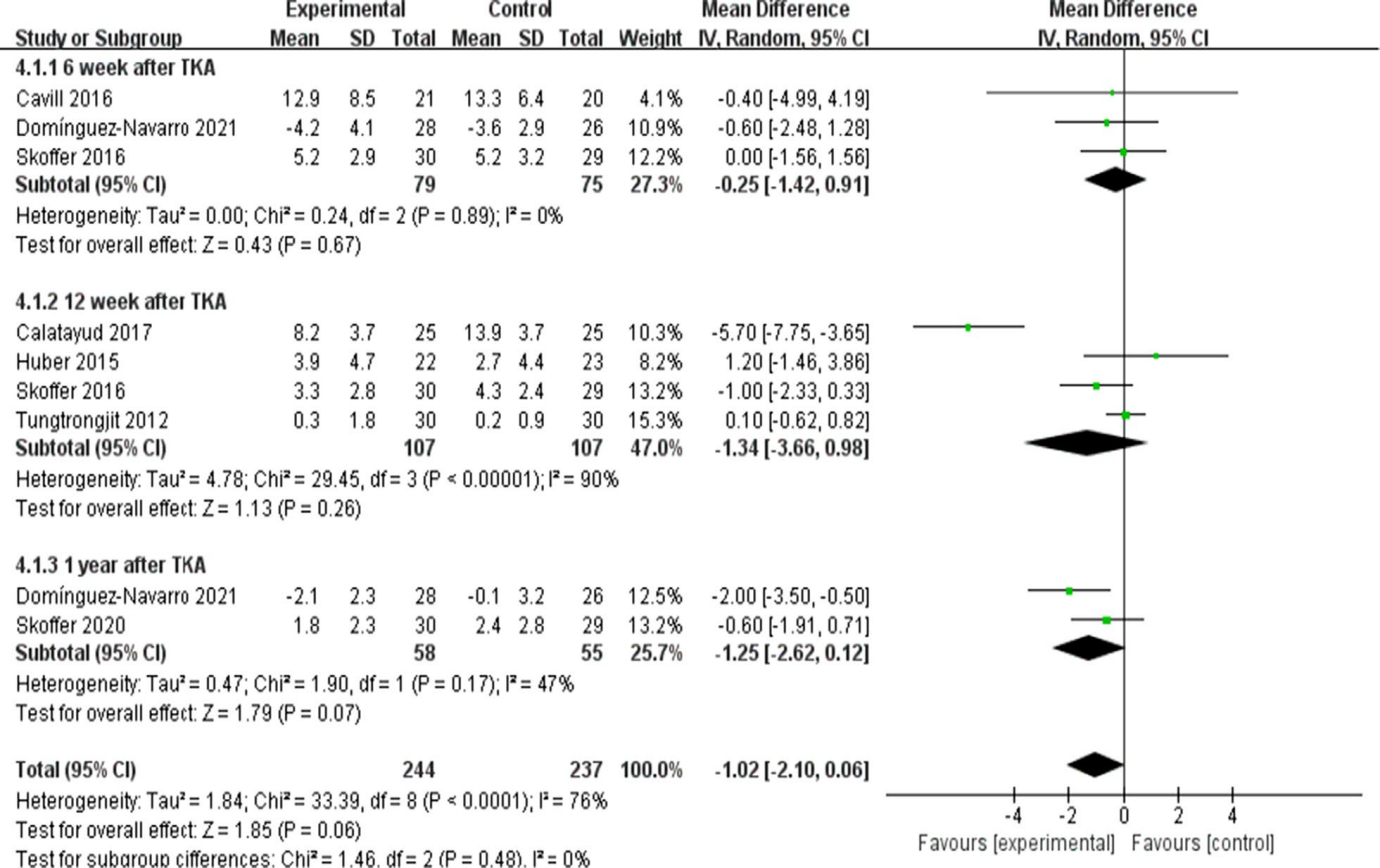
A forest plot diagram showing the knee extension

On the whole, two trials [11, 26] (110 patients) presented information regarding knee ROM and eight trials [8, 10, 16, 19–22, 26] (404patients) presented the information regarding knee extension and seven trials [8, 10, 16, 20–22, 26] (368patients) presented the information regarding knee flexion. No significant difference received the identification between the two group for ROM at the 6th week after TKA [*I*^2^ = 0%, MD = 5.4, 95% CI (− 0.12, 10.93), *P* = 0.06)] (Fig. 5). For flexion and extension, data were extracted three times, Subgroup analysis was conducted data extraction was conducted at the 6th week, the 12th week and the 1 year after TKA. Statistical distinction was found between the two groups [*I*^2^ = 70%, MD = 3.8, 95% CI (0.6, 7.01), *P* = 0.02] (Fig. 6). Not any obvious distinction was reported for knee extension between the two groups [*I*^2^ = 76%, MD = − 1.02, 95% CI (− 2.10, 0.06), *P* = 0.06] (Fig. 7).

#### Timed-up-and-go (TUG) and 6-min walk

**Fig. 8:**
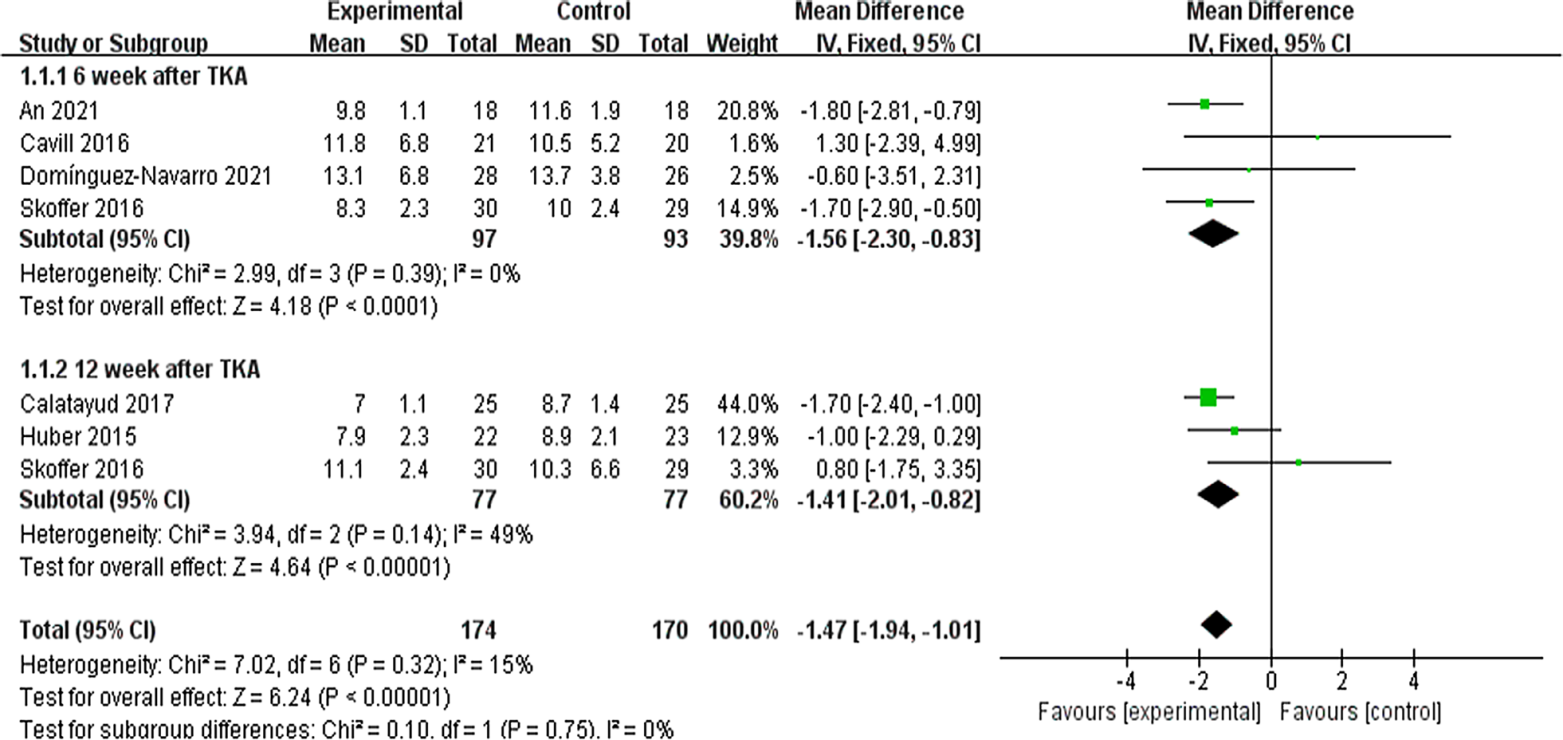
A forest plot diagram showing the time up and go

**Fig. 9:**
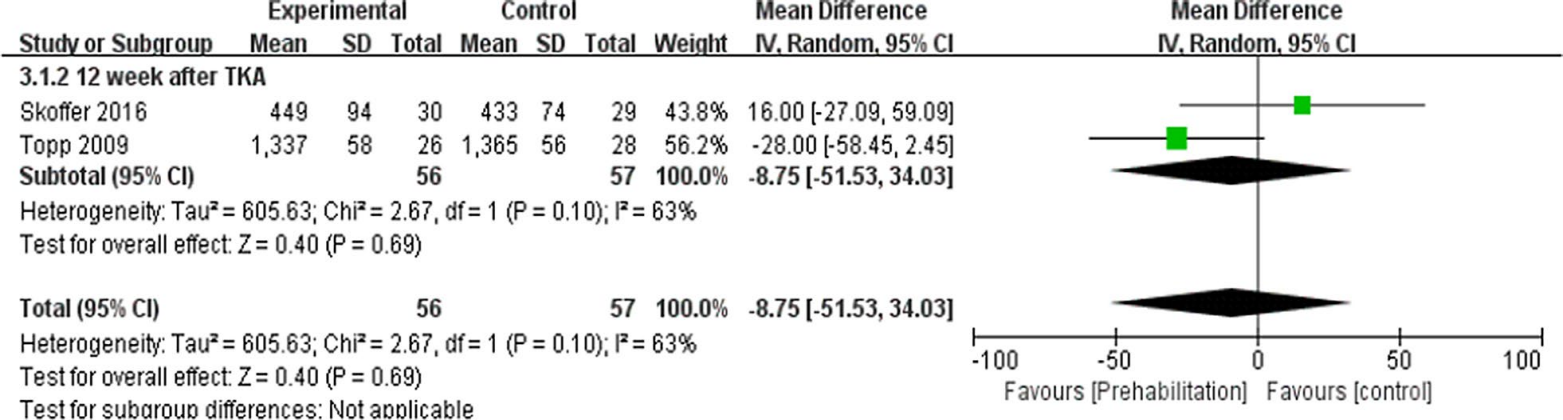
A forest plot diagram showing the 6-min walk

Six trials here [8, 10, 16, 19–21] (285 participants) provided data of TUG. In this study, data were extracted twice. Subgroup analysis was conducted at the 6th week and the 12th week after TKA. Noticeable difference was reported between two groups for TUG [*I*^2^ = 15%, MD = − 1.47, 95% CI (− 1.94, − 1.01), *P* < 0.01] (Fig. 8). Two trials [21, 28] (113 patients) presented the information of 6-min walk at the 12th week after TKA. No significant difference was reported between the two group in 6-min walk test [*I*^2^ = 63%, MD = − 8.75, 95% Cl (− 51.53to 34.03), *P* = 0.69] (Fig. 9).

#### Patient-reported functional outcomes

**Fig. 10:**
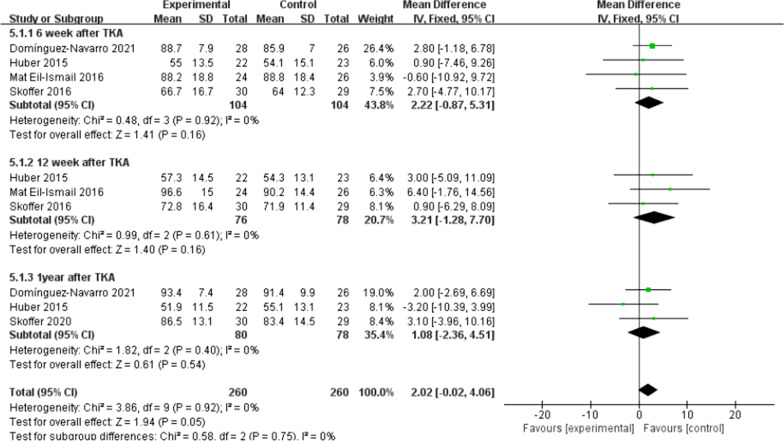
A forest plot diagram showing the KOOS symptoms

**Fig. 11:**
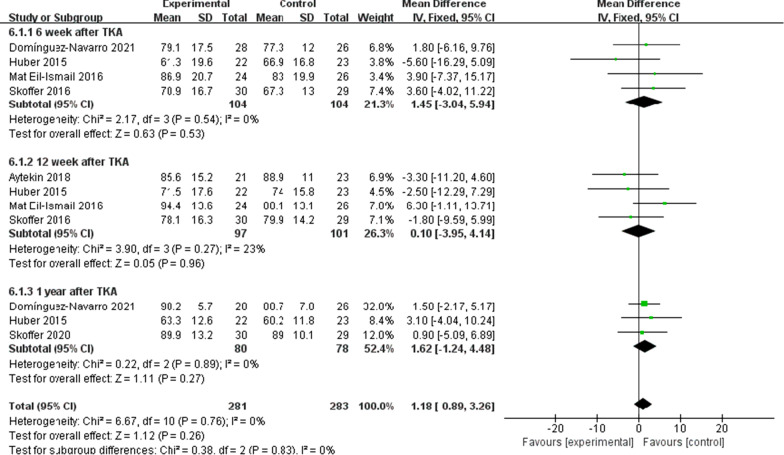
A forest plot diagram showing the KOOS pain

**Fig. 12:**
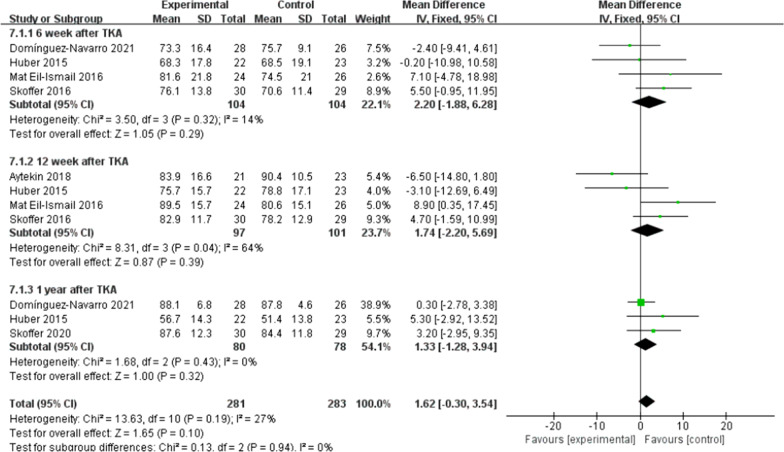
A forest plot diagram showing the KOOS function of daily living

**Fig. 13:**
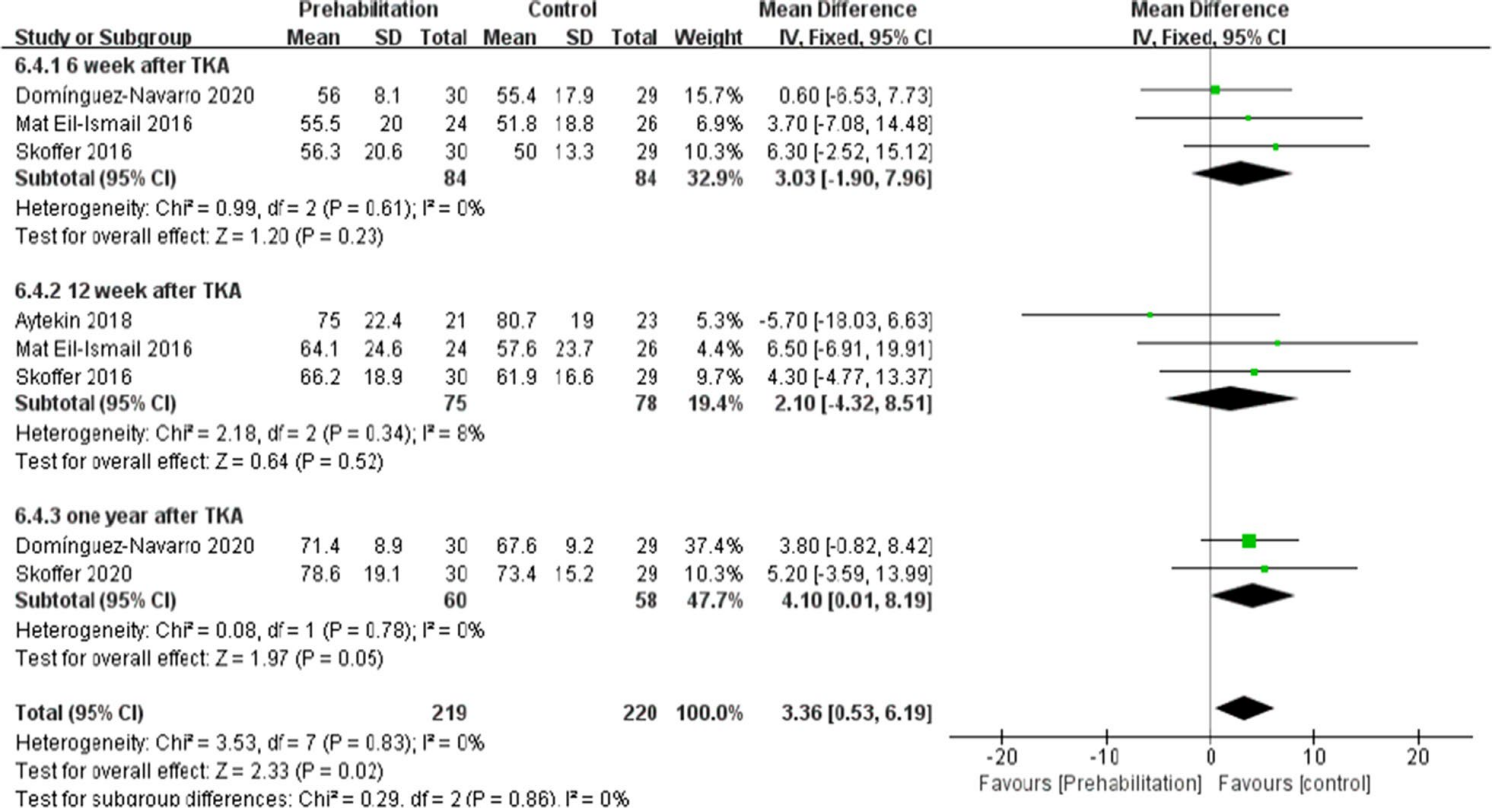
A forest plot diagram showing the KOOS knee-related quality of life

**Fig. 14:**
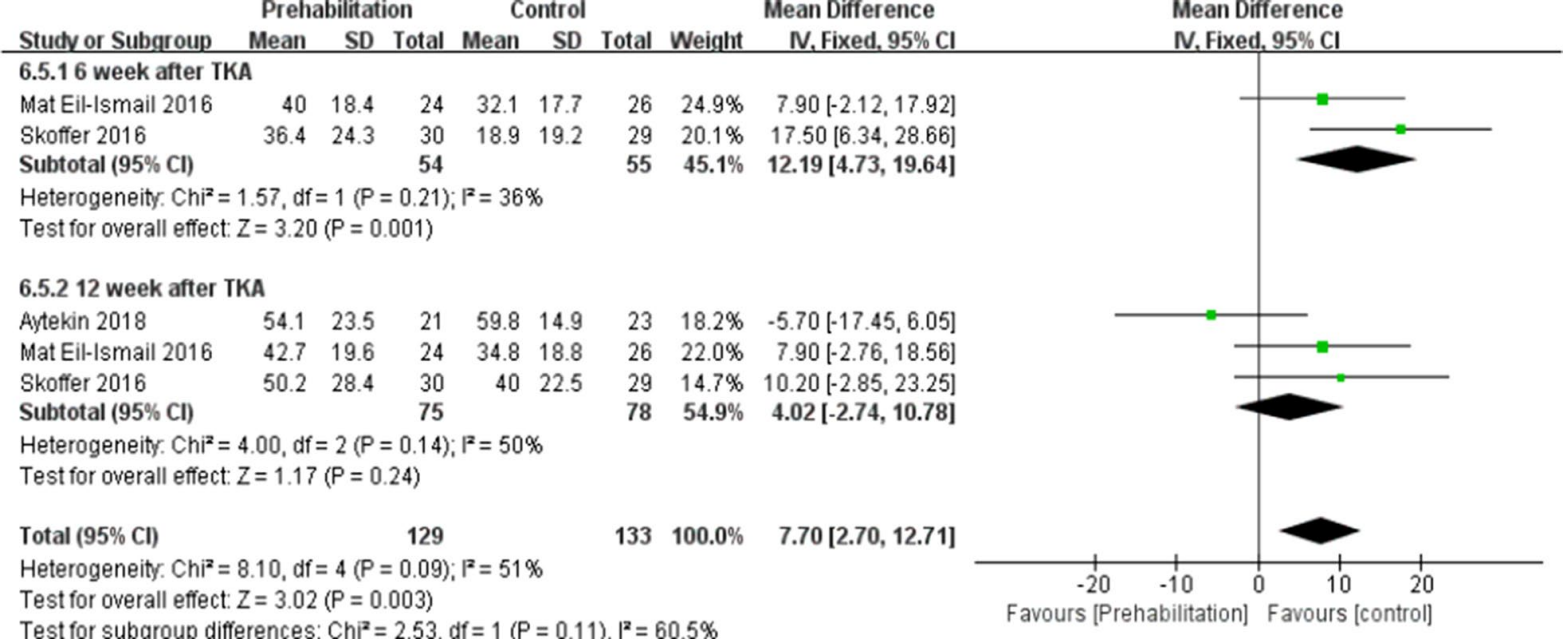
A forest plot diagram showing the KOOS function in sport and recreation

**Fig. 15:**
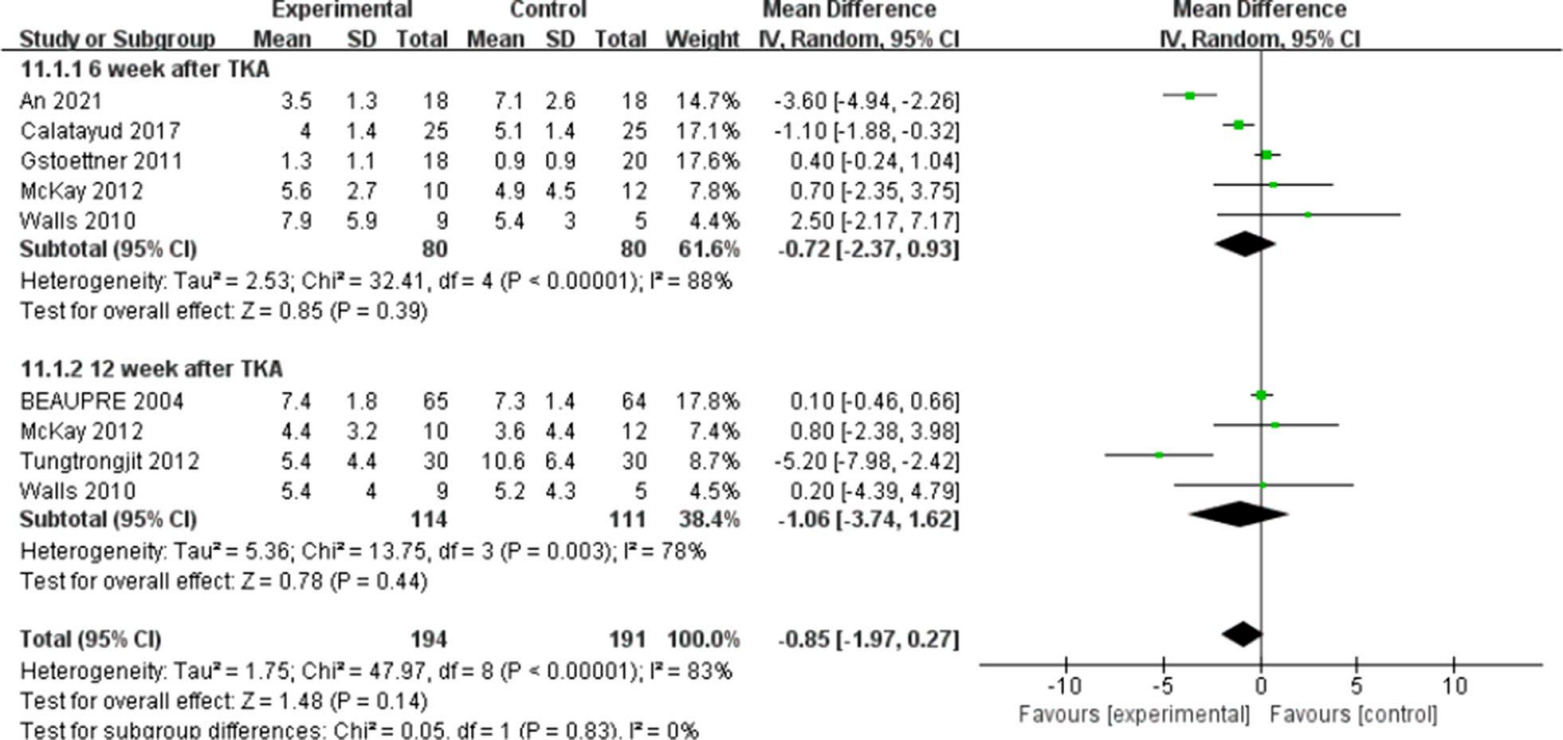
A forest plot diagram showing the WOMAC pain

**Fig. 16:**
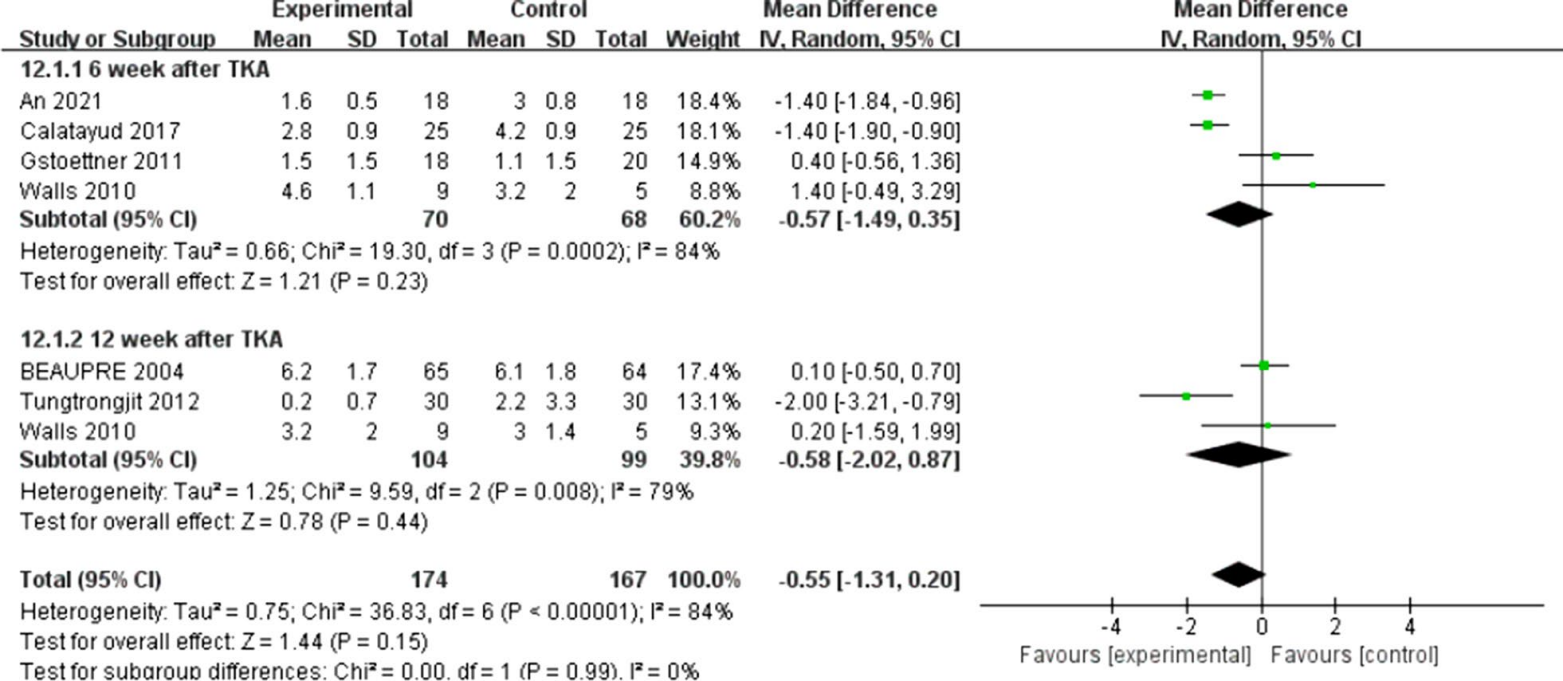
A forest plot diagram showing the WOMAC stiffness

**Fig. 17:**
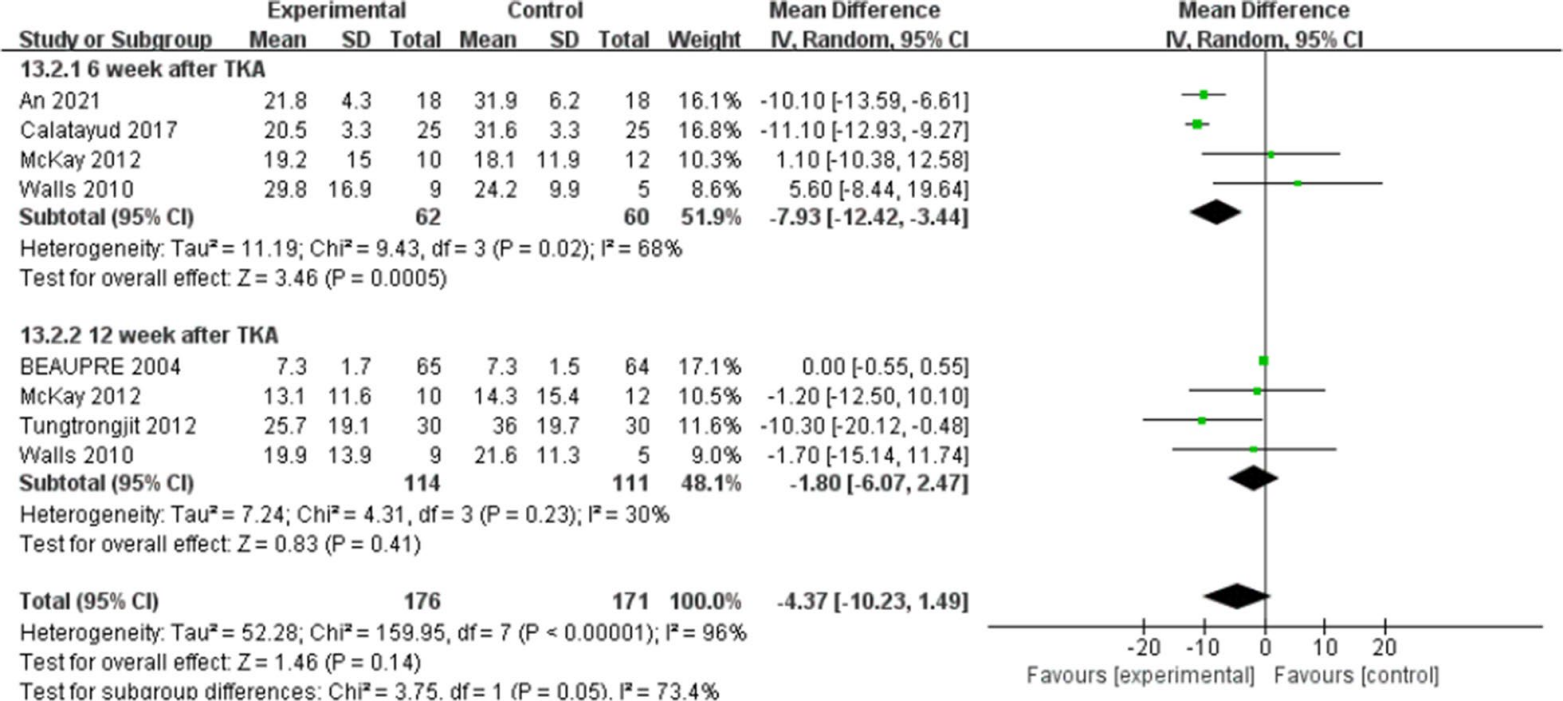
A forest plot diagram showing the WOMAC function

There were five trials [10, 11, 16, 21, 22] (267 patients) reported the data of KOOS symptoms, six trials [10, 11, 15, 16, 21, 22] (311 patients) reported the data of KOOS (knee-associated life quality, daily living function, pain), and four trials [10, 11, 15, 21] (198 patients) reported the data of KOOS functions in sports and recreation. Data were extracted three times, the subgroup analysis was conducted at the 6th week and the 12th week and one year after TKA during KOOS symptoms, pain, function of daily living, knee-related quality of life. There was no statistical difference between the two groups for KOOS symptoms [*I*^2^ = 0%, MD = 2.02, 95% CI (− 0.02, 4.06), *P* = 0.05] (Fig. 10). No statistical difference was identified between the two groups for KOOS pain [*I*^2^ = 0%, MD = 1.18, 95% CI (− 0.89, 3.26), *P* = 0.26] (Fig. 11). There was no statistical difference between the two groups for KOOS function of daily living [*I*^2^ = 27%, MD = 1.62, 95% CI (− 0.30, 3.54), *P* = 0.10] (Fig. 12). A statistical difference was found between the two groups for KOOS knee-related quality of life [*I*^2^ = 0%, MD = 2.87, 95% CI (0.23, 5.52), *P* = 0.03] (Fig. 13). For KOOS functions in sports and recreation, data were extracted twice. The subgroup analysis was conducted at the 6th week and the 12th week after TKA. A statistical difference was found between the two groups [*I*^2^ = 42%, MD = 7.51, 95% CI (3.37, 11.65), *P* < 0.01] (Fig. 14). There were seven trials [8, 19, 23–27] (349 patients) reported the data of WOMAC pain, six trials [8, 19, 23, 25–27] (311 patients) reported the data of WOMAC stiffness, six trials [8, 19, 23–26] (301 patients) reported the data of WOMAC function. The subgroup analysis was conducted at the 6th week and the 12th week after TKA. No statistical difference was identified between the two groups for WOMAC (pain, stiffness, function) (Figs. 15, 16, 17).

#### The length of hospital stay

**Fig. 18:**
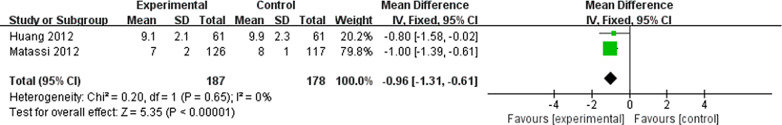
A forest plot diagram showing the length of hospital stay

Of the 19 trials, two trials [9, 17] (365 patients) reported the data of length of hospital stay. The pre-habilitation group showed a shorter length of hospital stay when compared with the control [*I*^2^ = 0%, MD = − 0.96, 95% Cl (− 1.31, − 0.61), *P* < 0.001] (Fig. 18).

## Discussion

The efficacy of preoperative rehabilitation on functional recovery for patients having received TKA remains controversial [17, 29]. Previous systematic reviews [12] reported that compared with the control, preoperative rehabilitation had a consistent functional recovery effect in patients having received TKA. Recently, preoperative rehabilitation has still aroused huge attention, and considerable studies were conducted on the effect of preoperative rehabilitation for functional recovery after TKA [8, 15, 16, 21]. Accordingly, the meta-analysis of the present study updated the literature to further assess the effect of preoperative rehabilitation on postoperative functions for patients having received TKA. This study made a summary of evidence from 19 randomized controlled trials which provided a clearer pole of preoperative rehabilitation for patients with TKA. According to the results of this study, preoperative rehabilitation was effective in reducing the length of hospital stay. It could be effective in improving knee flexion, TUG, KOOS (knee-related quality of life and functions in sports and recreation). However, it did not alter among pain, 6-min walk, ROM, knee extension, KOOS (symptoms, pain, function of daily living), WOMAC (pain, stiffness, function) following TKA. There was a certain heterogeneity among the included studies, which may be due to the different evaluation scales held by different researchers.

Pain was the primary outcome in the meta-analysis here. As it was uncovered from this study, preoperative rehabilitation did not increase postoperative pain following TKA in terms of the VAS scores either at the 6th week or the 12th week postoperatively, which was consistent with precedent studies [30]. Pain acts as the main symptom of knee OA and a key determinant of knee extension and flexion in knee OA. Thus, it has become one of the main problems to be solved by TKA. Such a study further showed that OA who had never exercised thought exercise might damage joints. However, preoperative rehabilitation is capable of reducing this fear, helping find ways to cope with pain, and maintaining exercise levels after surgery to improve their quality of life in depth [8].

Moreover, the knee range of motion is a vital indicator of postoperative functional recovery. As illustrated by Skoffer et al. [21] as opposed to the control, the 4-week preoperative progressive resistance training failed to significantly mitigate the knee flexion and extension at the 6th week and the 12th week postoperatively. As reported by Mat et al. [11] no significant difference in ROM was identified in the two groups. However, An [19] indicated that preoperative tele-rehabilitation yielded improvement in the knee flexion at the 6th week postoperatively. In the meta-analysis here, the subgroup analysis was conducted at the 6th week and the 12th week after the surgery, and the results complied with the mentioned findings. The meta-analysis of the present study showed that compared with the control, the preoperative rehabilitation group has no improvement in knee ROM and knee extension. Statistical difference was found between the two groups in the knee flexion. Many factors are found to affect knee ROM (e.g., implant design, the surgical technique used, preoperative ROM, knee kinematics, associated perioperative complications and postoperative rehabilitation compliance), which all impact knee flexion after TKA [31]. However, a single factor (e.g., preoperative rehabilitation) has little impact on postoperative knee ROM [11]. Therefore, large sample and high-quality randomized controlled trial should be carried out to verify the effect of preoperative rehabilitation on knee range of motion in the future.

According to the meta-analysis here, the subgroup analysis was conducted at the 6th week and the 12th week after TKA, indicating that compared with the control, the rehabilitation group was preoperatively better in TUG. Skoffer et al. [21]showed that the TUG was better in the preoperative rehabilitation group than the control at the 6th week and the 12th week postoperatively. Calatayud et al. [8] also demonstrated that the 8-week preoperative high-intensity strength training improved TUG after TKA. The ability to walk refers to a basic ability in daily life, as well as a predictor of mobility and functional ability. The mentioned result further supports the conclusion that the Timed Up and Go test complied with the theory of preoperative rehabilitation [28]. The 6-min walk test measured the maximum walking distance covered in 6 min. As indicated from the result of the meta-analysis here, the preoperative rehabilitation group had consistent results on the 6-min walk compared with the control. Topp et al. [28] and Skoffer et al. [21] reported that no significant difference in 6-min walk was reported between the preoperative rehabilitation group and the control for patients following TKA. This result may be attributed to the strength of the quadriceps, indicating that the stronger the quadriceps, the longer the 6-min walk will be [28].

For the outcome of self-reported physical function, compared with patients allocated to the control group, no significant improvement was observed except for the KOOS (sport and knee-associated quality of life subscale) on TKA patients who received preoperative rehabilitation. Skoffer et al. [21] reported that no differences were identified between the groups in KOOS, except for the KOOS sport subscale in favor of the preoperative rehabilitation group. According to Mat et al. [11] a noticeable distinction was reported in symptoms and ADL function, but no significant difference was found for other KOOS subscales. Aytekin et al. [15] also declared no significant differences within both groups in KOOS. Calatayud et al. [8] reported that no improvement for WOMAC function score was found in preoperative rehabilitation group. Likewise, Rooks et al. [32] also demonstrated that no significant difference in WOMAC function score between the preoperative rehabilitation group and the control group following TKA. All the included articles indicated that both groups of patients had significant improvement in patient-reported functional outcomes after TKA, not unrelated to whether they underwent preoperative rehabilitation or not. We found that the different programs, intensity and duration of preoperative rehabilitation in across enrolled studies might result in the heterogeneity of our outcomes. An et al. [19] declared that preoperative telerehabilitation could improve WOMAC functional results after TKA. Therefore, the effect of preoperative rehabilitation on postoperative function of TKA patients remains uncertain. In addition, Paravlic et al. [33] reported that home-based motor imagery intervention can improve functional performance after total knee replacement in the short term without increasing patients' pain. Motor imagery refers to the mental representation of body movements without obvious body movements, which can effectively improve the performance of sports [34]. A systematic review shows that motion imagery is effective in the treatment of strength enhancement, pain reduction, and improved physical activity in patients undergoing TKA [35]. The intervention time of preoperative rehabilitation is generally 4–8 weeks. Therefore, whether the preoperative rehabilitation combined with motor imagery has a positive effect on the knee function of TKA patients is worth further exploring. Furthermore, our meta-analysis showed that preoperative rehabilitation could significantly shorten the length of hospital stay, which was in congruity with the results of another meta-analysis by Chen [36]. We know that the length of hospital stay is affected by numerous factors (e.g., the time of postoperative suture removal), so it cannot act as one of the effective indicators to assess the rehabilitation effect.

The systematic review and here meta-analysis of the present study are subject to several limitations. (1) The number of literatures in the subgroup analysis was small in the meta-analysis here, and the sample size of the respective study was small, thereby reducing the statistical ability of our meta-analysis. (2) Only English and Chinese publications were included in our meta-analysis. Accordingly, publication bias is inevitable. (3) Outcomes (e.g., complications, muscle strength and knee society score were not analyzed as impacted by the lack of data. (4) The preoperative rehabilitation protocol varied with the studies. Different preoperative rehabilitation protocol may cause higher statistical heterogeneity on postoperative functional outcomes (e.g., knee extension).

## Conclusion

Preoperative rehabilitation could effectively shorten the length of hospital stay. Our meta-analysis showed that preoperative rehabilitation had the similar effect on postoperative functional recovery following TKA compared with the control group. In short, high-quality randomized controlled trials (RCTs) are needed to determine the efficacy of preoperative rehabilitation on postoperative recovery following TKA.

## Supplementary Information

**Additional file 1.** PRISMA checklist.

## Data Availability

The authors declare that all the data supporting the findings of this study are available within the article and its supplementary information files.

## Abbreviations

AMSTAR: Assessing the methodological quality of systematic reviews
KOOS: The Knee Injury and Osteoarthritis Outcome Score
OA: Osteoarthritis
PRISMA: Preferred Reporting Items for Systematic Reviews and Meta-Analysis
ROM: Knee range of motion
SMD: Standardized mean difference
TUG: Timed-up-and-go
TKA: Total knee arthroplasty
VAS: Visual Analog Scale
WOMAC: Western Ontario and McMaster Universities Osteoarthritis Index
WMD: Weighted mean difference
