# Supplementary material for: The effects of preoperative rehabilitation on pain and functional outcome after total knee arthroplasty: a meta-analysis of randomized controlled trials
Source: J Orthop Surg Res. 2022 Mar 21;17:175. doi: 10.1186/s13018-022-03066-9 (PMC8935773; doi:10.1186/s13018-022-03066-9)
Supplement: Supplementary file 1 — Additional file 1. PRISMA checklist. [file 13018_2022_3066_MOESM1_ESM.doc]

| **Section/topic** | **#** | **Checklist item** | **Reported on page #** |
| --- | --- | --- | --- |
| **TITLE** | | |  |
| Title | 1 | The report is identified a meta-analysis. | 1 |
| **ABSTRACT** | | |  |
| Structured summary | 2 | The structured abstract includes background, Methods, Results and Conclusions. | 1 |
| **INTRODUCTION** | | |  |
| Rationale | 3 | Described in the introduction. | 2 |
| Objectives | 4 | Described in the introduction. | 3 |
| **METHODS** | | |  |
| Protocol and registration | 5 | The protocol is described in the introduction, we have registered in the Research Registry (Web address: https://www.researchregistry.com/browse-the-registry#registryofsystematicreviewsmeta-analyses/), and registration ID is: reviewregistry113. | 4 |
| Eligibility criteria | 6 | Described in the methods. | 4 |
| Information sources | 7 | Described in the methods. | 4 |
| Search | 8 | Described in the methods. | 4 |
| Study selection | 9 | Described in the methods. | 4 |
| Data collection process | 10 | Described in the methods. | 5 |
| Data items | 11 | Described in the methods. | 5 |
| Risk of bias in individual studies | 12 | Described in the methods. | 5 |
| Summary measures | 13 | Described in the methods. | 5 |
| Synthesis of results | 14 | Described in the methods. | 5 |

Page 1 of 2

| **Section/topic** | **#** | **Checklist item** | **Reported on page #** |
| --- | --- | --- | --- |
| Risk of bias across studies | 15 | Described in the methods. | 6 |
| Additional analyses | 16 | Subgroup analyses. | 6 |
| **RESULTS** | | |  |
| Study selection | 17 | Described in the results. | 6 |
| Study characteristics | 18 | Described in the results. | 7 |
| Risk of bias within studies | 19 | Described in the results. | 7 |
| Results of individual studies | 20 | Described in the results. | 7 |
| Synthesis of results | 21 | Described in the results. | 7 |
| Risk of bias across studies | 22 | Described in the results. | 7 |
| Additional analysis | 23 | Subgroup analyses results. | 8 |
| **DISCUSSION** | | |  |
| Summary of evidence | 24 | Described in the discussion. | 9 |
| Limitations | 25 | Described in the discussion. | 14 |
| Conclusions | 26 | Described in the conclsion. | 14 |
| **FUNDING** | | |  |
| Funding | 27 | This work was supported by Health Commission of Hunan Province of China (No. 202114021174). | 15 |

*From:*  Moher D, Liberati A, Tetzlaff J, Altman DG, The PRISMA Group (2009). Preferred Reporting Items for Systematic Reviews and Meta-Analyses: The PRISMA Statement. PLoS Med 6(7): e1000097. doi:10.1371/journal.pmed1000097

For more information, visit: **www.prisma-statement.org**.

Page 2 of 2
